# Suicidal behaviour and resilience-related factors among adolescents in Poland and war-affected Western Ukraine

**DOI:** 10.1186/s13034-026-01126-0

**Published:** 2026-06-28

**Authors:** Krzysztof Ostaszewski, Agnieszka Bielawska, Anna Borucka, Jakub Greń, Inna Haletska, Halyna Herasym, Larysa Klymanska, Milena Markiewicz, Iryna Mirchuk, Agnieszka Pisarska, Viktor Savka, Oksana Zelena, Maryna Klimanska

**Affiliations:** 1 Institute of Psychiatry and Neurology, Warsaw, Poland; 2 Ivan Franko National University of Lviv, Lviv, Ukraine 1, Universytetska St., 79000; 3 Lviv Polytechnic National Universitу, Lviv, Ukraine; 4 Drohobych Ivan Franko State Pedagogical University, Drohobych, Ukraine; 5 WSB Merito University in Gdańsk, Gdańsk, Poland

**Keywords:** Traumatic experiences, Suicidal behaviour, Resilience, Adolescents, Risk factors, Protective factors, Socio-ecological model, School survey

## Abstract

**Background:**

The purpose of the research was to verify compensatory and protective models of resilience [14, 27] , among adolescents from Poland and war-affected Ukraine. Guided by the socio-ecological model of resilience theory, we examined the relationship between several psychosocial risk and protective factors and suicidal behaviour among adolescents.

**Method:**

Data were collected via online self-report questionnaires in Autumn 2024 from 14 to 15 year olds in Western Ukraine (*N* = 2179) and Warsaw, the capital of Poland (*N* = 1154). The response rate achieved was 75% in Lviv, 73% in Drohobych and 70% in Warsaw. Suicidal behaviour was measured by a 3-item Suicidal Thoughts and Behaviours scale adapted from the MINI Kid (Sheehan et al. 2010) (α = 0.87 − 0.90). Four groups of risk factors were examined: 1/ exposure to traumatic experiences, with a 9-item index, and victimization measured by two single questions; 2/ mental health problems (symptoms of depression measured by the shortened 4-item CES-D scale, non-suicidal self-injuries measured by a single question, problems of conduct measured with a 12-item index, problematic internet use measured with the Internet Disorder Scale – Short Form (IDS9-SF); 3/ substance use (alcohol, drug, cigarette and tranquiliser use, all measured with single questions); and 4/ personality characteristics that increase vulnerability (impulsiveness, measured by the Barratt Impulsiveness Scale–Brief (BIS-Brief), sensation seeking measured by a 6-item scale). Three groups of protective factors were also measured: 1/ parental/ familial (parental management, family relationships measured using the Brief Family Relationship Scale); 2/ school (school bonding measured with a 6-item scale, sense of safety at school measured by a single question); and 3/ individual (religiosity, measured by two single questions, individual physical activity measured by a single question, positive perception of the future, and five key aspects of positive youth development (connection, confidence, character, caring, competences), measured with the Positive Youth Development (PYD-VSF) scale. Hierarchical linear regression was conducted to test models of resilience.

**Results:**

Descriptive analyses showed that significantly more teenagers in Warsaw (Poland) than in Lviv and Drohobych (Ukraine) had suicidal thoughts in the past 12 months (33.6% vs. 14.7%), had at some time planned suicide (35.5% vs. 16.9%), and had at some time attempted suicide (17% vs. 7.2%). In both countries, suicidal behaviours were significantly more prevalent among girls than boys. Further analyses also showed that adolescents in Warsaw were more exposed to significant risk factors than their peers in Lviv and Drohobych and, conversely, that youth in Lviv and Drohobych were more strongly protected against these risks than those in Warsaw. Regression analysis indicated that traumatic experiences, victimization, depressive symptoms, non-suicidal self-injury and the use of tranquilizers were associated with increased suicidal behaviour in both countries, whereas sensation seeking and drug use were associated with increased suicidal behaviour among Ukrainian youth. The results of regression showed also that a positive perception of the future and parental (on the part of the mother) management significantly reduced the risk of suicidal behaviour in both countries; however, a sense of safety at school proved to be a significant protective factor among Ukrainian adolescents, while self-confidence played that role in the Polish group. A block of four protective factors was associated with reduced risk of suicidal behaviour in both countries, supporting a compensatory model of resilience. Sense of safety at school moderated the relationship between traumatic experiences and suicidal behaviour in both countries, supporting a protective model of resilience, whereas self-confidence moderated this relationship in the Polish sample while parental management (on the part of the mother) moderated this relationship in in the Ukrainian sample. Overall, the regression model explained over 50% of the variance in suicidal behaviour in Ukrainian adolescents and 58.5% in Polish adolescents.

**Conclusions:**

This study supports the usefulness of resilience models, demonstrating that individual strengths, family resources, and a safe school environment can moderate the negative consequences of traumatic experiences on adolescent suicidal behaviour. A specific implication of these findings is the need for targeted interventions that strengthen family and school support systems to reduce the risk of suicidal thoughts and behaviours among adolescents.

**Supplementary Information:**

The online version contains supplementary material available at https://doi.org/10.1186/s13034-026-01126-0.

## Introduction

### Context of the war

As neighbouring countries in Central and Eastern Europe with similar cultural and educational contexts, Poland and Ukraine found themselves at the centre of a shared regional crisis; Ukraine as the direct target of military aggression, and Poland as a country indirectly affected by the consequences of the conflict—namely, mass immigration from Ukraine, particularly of women and children, as well as constant exposure to war-related content in the media and perceived security threats. The threat of war deepens the uncertainty of young people in Poland about their future. The context of war-related threats increases the risk of depression, disorientation, and a loss of a sense of security, which may contribute to suicidal behaviour among Ukrainian and Polish youth. The results of the Ukraine Adolescent Mental Health Study conducted in 2016-17 and 2023-24 suggest that exposure to war-related trauma was associated with PTSD symptoms, depressive symptoms, and suicidal and self-harming behaviour. Authors of this study suggest that the ongoing war in Ukraine poses a major challenge to the mental health, resilience, and development of young people and their families [48, 49]. Research consistently links traumatic experiences to an increased risk of suicide among adolescents [43].

### Risk and protection

Adolescence is a crucial time of emotional, social, and psychological change, making this age group particularly vulnerable to mental health problems, including suicidal behaviour. The mental health of adolescents has deteriorated worldwide following the Covid-19 pandemic, which has increased children’s and young people’s exposure to traumatic events, social isolation, and affective disorders [41]. One of the negative consequences of these problems is the increase in suicidal behaviour observed over the past few years among adolescents under the age of 19 in Poland [25]. The situation in Ukraine in this regard has been stable in recent years, but the suicide rates among Ukrainian youth reported by the WHO were high compared to those in other European countries [59].

A history of childhood adversity is strongly linked to depression, anxiety, suicidal thoughts, and emotional disorders, which are exacerbated by traumatic experiences, emotional neglect, and negative school experiences such as bullying and social exclusion [33]. Early exposure to any interpersonal violence from childhood to young adulthood doubles the short-term risk of suicide attempts compared to peers who have not faced such experiences [8]. The likelihood of suicidal thoughts may increase up to 2.5 times in young people who have experienced sexual, physical, or emotional violence. Sexual abuse alone is associated with a 4-fold increase in the probability of suicide being planned [1].

However, the path from traumatic experiences to suicidal behaviour is complex and influenced by numerous risk factors, such as substance use and negative societal experiences like peer violence [43]. While understanding these risks is important, identifying and strengthening protective factors is vital for suicide prevention among adolescents [20]. The successful or unsuccessful development of adolescents exposed to traumatic experiences and other risks depends largely on the internal assets and external resources available to them.

Research shows that resilience factors have a protective effect on mechanisms common to various mental health problems, such as depression, anxiety disorders and suicidal behaviours. Their presence indicates the existence of interconnected protective factors and systems. Among adolescents, key factors that reduce the risk of suicide are a sense of connection with parents and parental support, including good communication and a strong bond between mother and child [2, 10, 38], a strong bond with school and a sense of security in the school environment, and absence of cyberbullying [4, 29, 36, 60], positive thinking about the future [23], and self-confidence and coping skills [3, 36] .

### Theoretical framework

Although individual factors (such as coping- and crisis management skills) play an important role in reducing the risk of suicidal behaviour among adolescents, the phenomenon of resilience cannot be reduced solely to an individual trait. Resilience is often defined as a multidimensional process and/or protective mechanism that modifies an individual’s response to chronic stress, traumatic events, or adverse living conditions, promoting positive adaptation and healthy development [47]. Researchers studying adolescent resilience argue that it is linked to human development processes and specific social interactions [30]. This view is confirmed both in Bronfenbrenner’s ecological model [7], describing the systems within which human development takes place, and in empirical research that laid the foundations for research on resilience processes [18, 46, 57]. The ecological model describes the interactions between children and their immediate environments (family, school, and peers) and broader contexts (community, society, culture), as well as the interactions between these different environmental levels. This framework provides a rationale for incorporating a variety of risk and protective factors into resilience research to represent the ecology of human development. Expanding on Bronfenbrenner’s ecological model, Ungar , [56] proposes shifting the focus of resilience research from the individual to the processes of shaping the social and physical environment. His model provides a rationale for studying resilience processes across different countries and cultures while considering the diversity of participants’ physical, political, social, and cultural contexts.

In recent years, research interest in resilience has grown in response to the growing global threats such as natural disasters, pandemics, armed conflicts and terrorist attacks. An approach that treats resilience as a dynamic and multidimensional process involving multiple interacting systems is gaining increasing importance. These include systems operating within the individual (e.g., temperament, social skills, self-control), as well as external systems which involve family, peers, school, neighbourhood, community, culture, and political system [27, 32].

### Rationale, purpose and hypothesis

The purpose of the current study is to test compensatory and protective models of resilience among adolescents from Poland (Warsaw) and war-affected Western Ukraine (Lviv and Drohobych). These two models of resilience explain how positive factors operate to help adolescents overcome risks. In the compensatory model, positive factors have a direct beneficial effect on outcomes and offset the negative effects of risk factors. The protective model, on the other hand, describes factors that moderate the association between risk factors and negative outcomes, such that the effect of risk factors is weaker when protective factors are present [14, 18, 30]. These two types of factors are known in the literature under the three terms “protective factors”, “promotive factors” or “resilience factors” [32, 63].

Guided by the socio- ecological model of resilience [56], we examine the relationship between traumatic experiences and suicidal behaviour among adolescents, along with a range of psychosocial risks, including mental health problems, victimization, substance use, and personality characteristics associated with increased vulnerability, as well as protective factors representing individual, family and school characteristics. This analytic approach is supported both theoretically by the ecological model of human development [7], and empirically by studies demonstrating a variety of risk and protective factors influencing adolescent suicidal behaviours [43, 52].

In this study, we hypothesized that exposure to traumatic experiences, mental health problems, and substance use would be associated with increased suicidal behaviour in adolescents. We also hypothesized that among adolescents exposed to these risk factors, family management, positive perceptions of the future, self-confidence or other manifestation of positive youth development, positive family relationships, individual physical activity, and a sense of safety at school would contribute to a reduction in suicidal behaviour (a compensatory effect) and/or act as a buffer against suicidal behaviour (a protective effect).

## Methods

The current study, conducted in 2024, is part of an ongoing Polish-Ukrainian school-based research project running since 2016 (e.g., [40]). The broad purpose of the study is to analyse trends and factors in youth mental health problems and risky behaviours, including substance use, problematic Internet use, symptoms of depression, and suicidal behaviour. It also provides a basis for comparing data, some of which was collected under the conditions of a full-scale war.

### Participants and procedure

A parallel and simultaneous school-based survey was conducted in Lviv, Drohobych (Western Ukraine) and in Warsaw (Poland) (November-December 2024), using the same methodology. Participants in the survey were secondary school students aged 14–15 selected through a classroom-based sampling approach, with parental consent obtained prior to participation. The survey was administered in classrooms by external data collectors, using an anonymous online questionnaire that students filled out on their smartphones or tablets. In both countries, the unit of randomisation was the school classroom. In Western Ukraine, 118 classes from 86 secondary schools were randomly selected; in Poland, 87 classes from 38 public and non-public secondary schools were drawn at random from three large districts of Warsaw.

The survey procedure was comparable in Warsaw, Lviv and Drohobych. School principals received an information letter describing the project, with a request for their consent for students to participate in the survey. Most principals in both countries agreed, however in Ukraine the principals of four schools and in Poland the principals of seven declined participation. After obtaining the principal’s consent, a letter was forwarded through the school to the parents requesting their consent to their child’s participation. In most schools, so-called passive consent was used, but in a few schools, at the request of the school administration, the procedure of active parental consent was applied. In Ukraine, all parents gave their consent, whereas in Poland parents of 262 students did not consent to participation. In addition, the students participating in the study answered the first question in the online survey: *Do you confirm that you have read the survey description and agree to participate in it?* (Yes or No). If the student chose “No,” further participation was not possible. The Bioethics Committee of the Institute of Psychiatry and Neurology approved the survey procedure.

The response rate in Ukraine was (75% in Lviv, 73% in Drohobych). The slightly lower response rate in Warsaw (70%) was mainly due to the high number of parental refusals.

Due to missing data reaching 80% of the survey questions or more, inconsistencies and patterns suggesting a non-serious attitude to the survey 121 records were removed from the Warsaw database, and 250 from the Ukrainian database. Finally, the sample included 2,179 students from Ukraine (53% female, 3.4% non-binary) and 1,154 students from Warsaw (52.4% female, 3.4% non-binary), for a total of 3,333 students (52.9% female, 3.4 non-binary) across both countries. The mean age of respondents in Ukraine was 14.3 years (*SD* = 0.53), while in Poland it was 14.7 years (*SD* = .80). The difference between the mean age in the two countries proved to be statistically significant (*t*(1687.05) = -13.409; *p* < 0.001, Cohen’s d =-0.55). In both countries, the majority of participants lived with both parents, although this was significantly more common in Ukraine (80.9%, compared to 72.2% in Poland, *p* < 0.05). Table 1A (supplemental material) shows the sociodemographic characteristics of the participants across the two countries.

### Measures

Dependent variable. The Suicidal Thoughts and Behaviours scale adapted from the MINI Kid Mini International Neuropsychiatric Interview for Children and Adolescents as part of the earlier EZOP II research project [39, 51] was used as the dependent variable. The adaptation of the MINI Kid questionnaire was part of a comprehensive, nationwide EZOP II study conducted in Poland, which included a sample of 2,000 children and adolescents aged 7 to 17. All items were carefully translated using a back-translation and harmonisation procedure by an interdisciplinary research team consisting of an experienced clinical psychiatrist, a sociologist, and a pedagogue, and were subsequently verified in a pilot study [34].

The Suicidal Thoughts and Behaviours scale consists of three questions assessing the frequency of suicidal thoughts (*Have you ever had suicidal thoughts in the last 12 months?*), suicide plans (*Have you ever in your life seriously thought about taking your own life or planned how to do it*?) and suicide attempts (*Have you ever in your life attempted suicide?*). Frequency of thoughts and plans was assessed on 4-point Likert scale (1 = did not happen, 4 = yes, often). Frequency of attempts was assessed on similar 4-point Likert scale (1 = did not happen, 4 = yes, three times or more). Although the scale includes a question (about suicidal thoughts) with a different time frame than the other two questions (about plans and attempts), we decided to combine them into a single scale for conceptual and empirical reasons. The question about suicidal thoughts is a key component of the clinical assessment of both suicide risk in ICD-10 and suicidal behaviour in DSM-5. Therefore, current suicidal ideation should be considered when measuring suicidal behaviour in adolescents.

The use of questions about suicidal thoughts within a shorter time frame has also empirical justification. The frequency of suicidal thoughts in the last 12 months was strongly positively correlated with the frequency of suicidal plans and suicide attempts over the entire lifetime. In the entire sample, Pearson’s r coefficient was .774 between past 12 months’ thoughts and lifetime plans and .606 between thoughts and attempts. We also considered that suicidal behaviour among 14-15-year-olds in the context of their entire lives and in the context of the past year does not differ as much as it does among adults.

The internal reliability of the scale was high (α = 0.86 in Ukraine and 0.87 in Poland). The theoretical validity was also adequate, as shown by the moderate or high positive correlation with other measures of mental health problems in both countries (*r*= 0.463 in Ukraine and *r*= 0.558 in Poland with Symptoms of Depression scale; *r*=0.536 in Ukraine and *r*= 0.613 in Poland with the non-suicidal self-injury measure). The mean score for the suicidal behaviour scale in Ukrainian sub-sample was 1.20 (SD = 0.51, skewness = 3.44), in the Polish sub-sample 1.50 (SD = 0.80, skewness = 1.72), and in the total sample 1.30 (SD = 0.64, skewness = 2.58). Although these skewness statistics for the outcome variable are not ideal, they fall within the range of 0 to 3, which makes them acceptable for use in hierarchical linear regression analyses.

The independent variables consisted of four groups of risk factors: 1/ exposure to traumatic experiences and victimization, 2/ mental health problems (symptoms of depression, non-suicidal self-injures, conduct problems, problematic Internet use), 3/ substance use (alcohol, drugs, cigarette and tranquilisers), 4/ personality characteristics associated with increased vulnerability (impulsiveness, sensation seeking). Protective factors were grouped into three categories: 1/ parental/familial factors (parental management, family relationships), 2/ school factors (school bonding, sense of safety at school) and 3/ individual factors (religiosity, individual physical activity, positive perception of future, and five key aspects of positive youth development (connection, confidence, character, caring, competences).

The choice of independent variables was guided by the socio-ecological models [7, 56], and informed by a substantial body of research assessing risk factors for suicidal behaviours [8, 33, 43] and protective factors in different adolescent adaptive systems, including individuals, families, schools and communities ([4, 10, 17, 23, 29, 32, 36, 38, 60].

The majority of questions and scales used for measuring independent variables (including single questions on victimization, substance use, sense of safety at school, family management and scales: the Barratt Impulsiveness Scale–Brief (BIS-Brief), Internet Disorder Scale (IDS9-SF), scale on School Bonding, the Brief Family Relationship Scale, shortened CES-D scale on depressive symptoms, and the scale on Sensation Seeking) were already used and harmonised for the previous Polish-Ukrainian school surveys [24, 40, 50]. For the purposes of this study, two new instruments were developed in Polish and Ukrainian—a Traumatic Experiences Index and a Conduct Problems Index—using items from the Polish version of the MINI Kid, which was developed as part of the earlier EZOP II research project [39]. Furthermore, with the permission of The Tufts University Institute [55], the Positive Youth Development (PYD-VSF) scale was adapted into Polish and Ukrainian. The wording of the questions and the psychometric properties of the PYD-VSF scale were examined in small-scale pilot studies conducted among both Polish and Ukrainian adolescents. In the case of the PYD-VSF scale, this was already the second adaptation to Polish conditions, which was harmonised with the adaptation to Ukrainian conditions for the purposes of this study. The first adaptation of this scale into Polish was carried out by Brałóg [5]. Table 1 presents the independent variables, including the number of items in each measure, means and standard deviations, Cronbach’s alphas (where appropriate), a sample item from each measure, and the source of the measure or source of the adaptation.

**Table 1:** Descriptive statistics and measures of the independent variables

Variables (No of items)	Ukrainian sample		Polish sample		Sample item (type of scale used)	Source
	M(SD)	α	M(SD)	α		
Risk factors: exposure to trauma						
Traumatic experiences index; adapted from the MINI Kid (9)	0.80 (1.26)	NA	1.49 (1.60)	NA	*Have you ever experienced anything truly terrible/traumatic in your life*,* e.g. flood*,* hurricane*,* earthquake?* 0 =‘no’, 1 = ‘yes’	Ostaszewski et al., [39], Sheehan, [51]
Victimization including violence at school and cyberbullying (2)	1.17 (0.43)	*r*=0.31	1.38 (0.62)	*r*=0.27	*Have you ever been in this situation: one or more people from your school*,* class or online community*,* for an extended period*,* regularly bullied you using/via the Internet or a cell phone in such a way that you suffered and found it difficult to defend yourself?* 4-point Likert 1 - ‘did not happen’ to 4 - ‘it happened 4 times or more’	Pyżalski [44]
Risk factors: Mental health problems						
Symptoms of depression; shortened CES-D scale (4)	1.86 (0.73)	0.85	2.11 (0.83)	0.88	*In the last 7 days how often have you felt sad?* 4-point Likert, 1 - ‘never or rarely’ to 4 - ‘all the time’	Radlof [45]
Non-suicidal self-injury (1)	1.34 (0.84)	NA	1.94 (1.29)	NA	*Have you ever in your life self-harmed your body without suicidal intent?* 4-point Likert, 1 - ‘no’ to 4 – ‘yes, three or more times’	Makowska and Gmitrowicz, [28]
Conduct problem index; adapted from the MINI Kid (12)	1.12 (1.59)	NA	1.57 (1.86)	NA	*Have you hit or injured someone (on purpose)?* 0 =‘no’, 1 = ‘yes’	Sheehan et al., [39, 51]
Problematic internet use, measured by Internet Disorder Scale – Short Form (IDS9-SF) (9)	1.78 (0.74)	0.87	2.08 (0.90)	.90	*Do you feel preoccupied with your online behaviour 5-point Likert scale from 1 = “never” to 5 = “very often"*	Klimanska et al., [24], Pontes, [42]
Risk factor: Substance use						
Alcohol use adapted from the ESPAD survey (1)	1.33 (0.78)	NA	1.43 (1.01)	NA	*How many times (if at all) in the last 30 days have you had any alcoholic beverage (e.g. beer*,* wine*,* vodka*,* cognac*,* whiskey)?* 7-point scale 1 - ‘I did not drink’ to 7 - ‘20 times or more’	ESPAD Group, [13]
Drug use (1)	1.07 (0.43)	NA	1.84 (1.32)	NA	*Did you happen to use drugs between last September and now?* 5-point Likert, 1 = ‘did not happen’ 5 = ‘more than a dozen times’.	Wolniewicz-Grzelak and Ostaszewski, [58]
Cigarette use traditional and e-cigarettes (2)	1.14 (0.54)	*r*=0.49	1.48 (1.03)	*r*=0.67	*Do you smoke traditional cigarettes?* 5-point Likert, 1 = ‘no” 5 = ‘yes, everyday’.	Research team
Tranquilizers use (1)	1.45 (0.96)	NA	1.65 (1.23)	NA	*Have you taken any tranquilizers or sleeping pills since last September? 5-point Likert*,* 1 = ‘did not happen’ 5 = ‘yes*,* more than a dozen times’.*	Wolniewicz-Grzelak and Ostaszewski, [58]
Risk factor: personality characteristics that increase vulnerability						
Sensation seeking (4)	1.61 (0.84)	0.82	2.01 (1.00)	0.85	*In the last 6 months*,* how often have you done anything dangerous only to the thrill it causes?* 5-point Likert 1 - ‘never’ to 5 - ‘very often’	Frączek and Stępień, [16]
Impulsiveness measured by Barratt Impulsiveness Scale–Brief (BIS-Brief) (8)	2.18 (.47)	0.65	2.51 (0.59)	0.81	*I act without thinking*,* 4-point Likert*,* 1 - ‘never or rarely’ to 4 - ‘almost all the time or all the time’*	Steinberg et al., [53]
Protective factors. parental/ familial						
Parental management (mother) (1)	3.65 (1.21)	NA	3.25 (1.22)	NA	*How often do you talk to your mom when you have a problem?* 5-point scale 1 - ‘never’ to 5 -‘always’	Ostaszewski, [37], Zimmerman, [62]
Parental management (father) (1)	3.03 (1.36)	NA	2.59 (1.26)	NA	*How often do you talk to your dad when you have a problem?* 5-point scale 1 - ‘never’ to 5 -‘always’	Ostaszewski ,Zimmerman, [37, 62]
Family relationships measured by Brief Family Relationship Scale (12)	3.05 (0.62)	0.91	2.82 (0.62)	0.91	*In our family*,* we really help and support each other)* 4-pt Likert 1 = ‘definitely no’ 4 = ‘definitely yes’	Fok et al., [15]
Protective factors. school						
Sense of safety at school (1)	1.88 (0.84)	NA	1.71 (0.62)	NA	*Do you agree with the statement: I feel safe at my school.* 4-pt Likert 1 = ‘definitely no’, 4 = ‘definitely yes’	Research team
School bonding adapted from Flint Adolescent Study (6)	2.86 (0.60)	0.79	2.86 (0.52)	0.75	*I feel connected to my school*,* 4-point Likert*,* 1 = ‘definitely no’*,* 4 = ‘definitely yes’*	Ostaszewski,Zimmerman, [37, 62]
Protective factors. Individual						
Self-confidence: measured by PYD-VSF subscale (3)	3.55 (1.11)	0.88	3.09 (1.07)	0.87	*I am happy with myself most of the time*, 5-point scale 1- ‘strongly disagree’ to 5 -‘strongly agree’	The Tufts University Institute, [55]
Positive future perception (1)	7.43 (2.15)	NA	6.73 (2.49)	NA	*How do you think about your future?*10-point scale from 1 - ‘pessimistically’ to 10 - ‘optimistically’	Research team
Individual physical activity (1)	2.27 (1.11)	NA	2.40 (1.16)	NA	*How many hours per week do you spend on: cycling*,* roller-*,*skateboarding*,* swimming*,* running*,* climbing*,* gym or other forms of personal physical activity?* 5-point scale from 1 - ‘0 h’ to 5 - ‘8 h or more’	Bobrowski [6]
Religiosity (2)	2.64 (0.91)	*r* = 0.61	2.01 (0.96)	*r* = 0.64	How often do you go to church? 4-point Likert 1 = ‘never’ 4 = ‘once a week or more often’	Research team

M: mean; SD: standard deviation, α – Cronbach’s alpha, NA - not applicable

Sociodemographic variables included gender, age, and family structure. Gender was coded as 1 = males, 2 = females and 3 = non-binary. Participants’ ages ranged from 12 to 18 with 14- and 15-year-olds forming the largest group in the sample. Family structure responses included ‘living with both parents,’ ‘living only with father,’ ‘living only with mother,’ ‘living with one of the parents,’ ‘living with stepmother/stepfather,’ and ‘living with another person.’ Based on these responses, a dichotomous variable was constructed where 1 = ‘living with both parents’ and 2 = ‘not living with both parents’. The country in which the research was conducted was marked as follows: Ukraine (Lviv and Drohobych) = 1, Poland (Warsaw) = 2.

### Analysis

Bearing in mind the main goal of our research, we chose hierarchical linear regression for our analyses, as it allows for empirical verification of the theoretical assumptions in the concept of resilience. In line with the statistical criteria adopted in research on resilience, the compensatory model of resilience was tested by examining the unique, direct effects in a multiple regression analysis, while the protective model was tested using an interaction term in multiple regression [14]. Alternative analytical models, such as logistic regression or GENLIN, were also considered due to certain deviations from the normal distribution in our variables; however, these methods do not provide such clear answers to our research hypotheses. For full transparency, it should be noted that our analytical choice was a compromise between our research goals and the fully satisfying the requirements of linear regression.

Hierarchical multiple regression was used with suicidal behaviour as the dependent variable. A six-step hierarchical multiple regression was conducted for the total sample, and the Ukrainian and Polish sub-samples. The first step included four or three sociodemographic variables entered as a block (country – only for the whole sample, gender, age and family composition), then the risk factors were entered in the following order: traumatic experiences and victimization (second step), mental health problems (third step), and substance use and personality characteristics (fourth step). All the protective factors were entered as a block in the fifth step (to test the compensatory model of resilience), and the sixth step included several interaction terms with a traumatic experiences index by significant protective factors (to test the protection model of resilience). Measures used to create interaction terms were centred to facilitate interpretation of the interaction.

All measures which moderately correlated with the dependent variable were used in the multiple regression analyses. None of the correlations exceeded .6, which could expose the regression analyses to the negative consequences of multicollinearity. After controlling for sociodemographic variables, a selection procedure based on F statistics was used to find the best model predicting suicidal behaviour among adolescents. As a result of this process, several variables were excluded from the final model, as they were found to be insignificant in multivariate analyses or did not significantly raise the model’s explained variance. These included conduct problems, problematic Internet use, impulsiveness, school bonding, religiosity, parental management (Father), family relationships, PYD- VSF subscales (connection, character, caring, competences), alcohol use, cigarette/e-cigarette use, individual physical activity).

*Missing data.* Multiple imputation (MI) was used to handle missing data. All independent variables included in the final analysis (excluding categorical sociodemographic variables) were incorporated into the MI model.

Additional analysis. Additionally, hierarchical regression analysis was repeated with the block of protective factors entered in equations (as a second step) before risk factors blocks, to determine how much variance the protective factors explained without first accounting for risk factor variance. To better explain the differences in the prevalence of suicidal behaviour among Polish and Ukrainian adolescents, we also conducted comparative analysis of the intensity of significant risk factors and protective factors for suicidal behaviour in both sub-samples.

## Results

*Descriptive analyses.* Suicidal behaviours. The detailed prevalence of suicidal thoughts, plans and attempts by gender and country is shown in Table 2A (Supplemental Material). Significantly more adolescents in Warsaw (Poland) had suicidal thoughts, plans and attempts compared to those in Lviv-Drohobych (Ukraine). In both countries, suicidal behaviours were significantly more prevalent among girls than boys. Prevalence of suicidal behaviour among non-binary teenagers was notably high; although this sub-group in both countries was small, rates of all three suicidal behaviours studied were two-to-three times higher than in the entire sample.

In both sub-samples most teenagers had never made suicidal attempts; however, about 7% of the Ukrainian and about 17% of the Polish study participants were at high risk of suicidal behaviour due to previous suicidal attempts.

*Traumatic experiences*. Detailed prevalence of single and complex traumatic experiences both by country sample is shown in Table 3 A (Supplemental Material). The most commonly reported single traumatic events in both countries were witnessing someone being killed or injured and exposure to traffic accidents. Among ongoing or repeated traumatic situations, serious conflicts at home and peer violence predominated. A cross-country comparison showed that significantly more teenagers from Warsaw (Poland) than teenagers from Lviv-Drohobych (Ukraine) were exposed to most of the traumatic events and situations studied.

### Multiple regression analyses

Full sample. The results of regression analyses are presented in Table 2. After controlling for sociodemographics, the multiple regression for suicidal behaviour at the entire group level indicated main effects for the blocks of traumatic experiences and victimization (F-change (2, 3326) = 561.35; *p* < 0.001), mental health problems (F-change (2, 3324) = 611.04; *p* < 0.001), substance use and personality (F-change (3,3321) = 29.99; *p* < 0.001), protective factors (F-change (4, 3317) = 27.30; *p* < 0.001), and traumatic experience by protective factor interactions (F-change (3, 3314) = 40.60; *p* < .001). Sociodemographic variables entered as a block were significant as a predictor across the group (F-variable (4,3328) = 92.85; *p*<0.001), indicating that being a non-binary person significantly increased the risk of suicidal behaviour (*p* < 0.05). The regression model for the entire group explained 55% of the variance.

**Table 2:** Hierarchical linear multiple regression results predicting youth suicidal behaviour, from sociodemographic variables, traumatic experiences, mental health problems other risks, and protective factors

	Total N = 3328			Ukrainian sample N = 2175			Polish sample N = 1150		
	Final B	R^2^	ΔR^2^	Final B	*R* ^2^	ΔR^2^	Final B	R^2^	ΔR^2^
Sociodemographic factors		0.100	0.100***		0.028	0.028***		0.092	0.092***
Gender (1 = boys, 2 = girls, non-binary = 3)	0.037*			0.025			0.045		
Family structure (1 = both parents, 2 = other)	0.005			− 0.014			0.026		
Age in years	0.014			0.002			0.028		
Location (1 = Ukraine, 2 = Warsaw)	0.026			----------			--------		
Traumatic experiences		0.327	0.227***		0.274	0.246***		0.320	0.228***
Trauma - index of experiences (0–9)	0.043***			0.040***			0.050***		
Victimization	0.102***			0.061**			0.128***		
Mental health problems		0.508	0.181***		0.441	0.166***		0.526	0.207***
Symptoms of Depression	0.131***			0.110***			0.161***		
Non-suicidal Self-injures (lifetime)	0.196***			0.182***			0.191***		
Individual Risk factors		0.520	0.013***		0.471	0.030***		0.530	0.008***
Sensation Seeking	0.031***			0.045***			0.014		
Tranquilizers Use	0.052***			0.050***			0.050***		
Drug Use	0.057***			0.131***			0.035		
Protective factors		0.534	0.015***		0.480	0.009***		0.562	0.028***
Parental Management (Mother))	− 0.031***			− 0.014*			− 0.064***		
Positive Perception of the Future	− 0.023***			− 0.013***			− 0.033***		
Self-Confidence (PYD -VSF subscale)	− 0.020*			− 0.008			− 0.045*		
Sense of Safety at School	− 0.025*			− 0.035***			− 0.021		
Risk x protective interaction		0.550	0.016***		0.501	0.021***		0.586	0.023***
Trauma x Parental Management (Mother)	− 0.012**			− 0.023***			− 0.005		
Trauma x Self-Confidence (PYD sub-scale)	− 0.017***			0.008			− 0.041***		
Trauma X Feeling Safe at School	− 0.046***			− 0.046***			− 0.056***		

Final B- unstandardized coefficients B at the final step,* R*^*2*^- the incremental R^*2*^, *ΔR2*- the R^*2*^change at each step of the regression analysis

Ukrainian group of participants. After controlling for sociodemographics, the multiple regression for suicidal behaviour in the Ukrainian group indicated main effects for the blocks of traumatic experiences (F-change (2, 2173) = 368.56; *p* < 0.001), mental health problems (F-change (2, 2171) = 322.94; *p* < 0.001), substance use and personality (F-change (3,2168) = 40.91; *p* < 0.001), protective factors (F-change (4, 2164) = 9.65; *p* < 0.001), and traumatic experience by protective factor interactions (F-change (3, 2160) = 30.38; *p* < 0.001). Sociodemographic variables entered as a block were significant as a predictor across the Ukrainian group (F-variable (3,2175) = 20.85; *p*<0.001). The regression model for this group explained 50% of the variance.

Polish group of participants. After controlling for sociodemographics, the multiple regression for suicidal behaviour in the Polish group indicated main effects for the blocks of traumatic experiences (F-change (2, 1148) = 191.93; *p* < 0.001), mental health problems (F-change (2, 1146) = 250.06; *p* < 0.001), substance use and personality (F-change (3,1143) = 6.34; *p*<0.001), protective factors (F-change (4, 1139) = 18.44; *p* < 0.001), and traumatic experience by protective factor interactions (F-change (3, 1135) = 21.21; *p* < 0.001). Sociodemographic variables entered as a block were significant as a predictor across the Polish group (F-variable (3,1150) = 38.56; *p*<0.001). The regression model for this group explained 58.5% of the variance.

Multiple regression results indicated that the risk factors representing exposure to traumatic experiences (traumatic experiences index, victimization), mental health problems (symptoms of depression, non-suicidal self-injuries), and use of tranquilizers were associated with an increased frequency of suicidal behaviour in the entire group and in both samples (Ukrainian and Polish). Drug use and sensation seeking emerged as significant risk factors for suicidal behaviour only in Lviv / Drohobych (Ukraine), and were not significant in the Warsaw (Poland) sub-sample.

All four protective factors were statistically significant in the full sample. Two of them—positive perception of the future and parental management (on the part of the mother) —were significantly associated with lower risk of suicidal behaviour across the entire group of participants and in the Ukrainian and Polish sub-samples, but the other two protective factors had different effects on the Polish and Ukrainian groups. In the Ukrainian group, feeling safe at school proved to be a significant protective factor against suicidal behaviour, while in the Polish group it was not found to be significant. Conversely, the PYD-VSF self-confidence subscale was only found significant in the Polish sub-sample.

The multiple regression model also included several interaction terms between traumatic experiences and protective factors. Results indicated that sense of safety at school significantly buffered the negative effect of traumatic experiences on the risk of suicidal behaviour in both Ukrainian and Polish adolescents (as well as in the full sample). Parental management (mother) buffered the negative effect of traumatic experiences in the Ukrainian group (and in the full sample) and self-confidence (measured by the PYD-VSF subscale) in the Polish sub-sample and in the entire group.

Figures 1A, 2A and 3A (Supplemental Material) illustrate significant interaction effects observed across the full sample. Figure 1A illustrates the relationship between the risk factor (exposure to traumatic experiences) and the severity of suicidal behaviour for low (one standard deviation below the mean), medium (mean) and high (one standard deviation above the mean) levels of parental management (talking to the mother when a problem arises). These results indicate that the association between traumatic experiences and the severity of suicidal behaviour is weakest among adolescents who reported the highest level of parental management by the mother. Figure 2A illustrates the relationship between the risk factor (exposure to traumatic experiences) and the severity of suicidal behaviour for low, medium (mean) and high levels of sense of safety at school. Similarly, the association between traumatic experiences and the severity of suicidal behaviour is weakest among adolescents who reported the highest level of feeling safe at school. Figure 3A illustrates the relationship between the risk factor (exposure to traumatic experiences) and the severity of suicidal behaviour for low, medium (mean) and high levels of the PYD-VSF subscale on self-confidence. Similarly, the association between traumatic experiences and the severity of suicidal behaviour is weakest among adolescents who reported the highest level of self-confidence. Figure 4A, 5A, 6A and 7A (Supplemental Material) illustrate significant interactions in the Polish and Ukrainian samples.

*Additional analysis.* With the block of protective factors entered first, the hierarchical multiple regression of adolescent suicidal behaviour indicated a stronger main effect for the block of four protective factors than in previous analyses: Full sample (F-change (4, 3325) = 225.67; *p* < 0.001; R^2^-change=.201), Ukrainian sub-sample (F-change (4, 2171) = 110.36; *p* < 0.001; R^2^-change=.164), Polish sub-sample (F-change (4, 1146) = 101.08; *p* < 0.001; R^2^-change=.237). The main effects for risk factors remained highly significant regardless of the order of entry.

Table 4A (supplementary material) presents a comparison of significant risk factors and protective factors in the two sub-samples. All eight risk factors relevant to suicidal behaviour compared using the Student’s t-test had a significantly higher mean among adolescents in Warsaw (Poland) than among Ukrainian adolescents, indicating a higher risk intensity. However, the effect sizes of the between-group comparisons were low-to-moderate (Cohen’s d ranged from − .19 to − .58). Our comparisons also clearly show that the means of three (out of four) protective factors were significantly higher among adolescents from Lviv and Drohobych (Ukraine) than in Warsaw, indicating greater protection among Ukrainian youth. In these comparisons, the effect sizes were also low (Cohen’s d ranged from .31 to .42). Only in the case of the feeling safe at school variable were no significant intergroup differences observed.

## Discussion

As expected, traumatic experiences, victimization, depressive symptoms and non-suicidal self-injures proved to be the strongest risk factors among adolescents in both Ukraine and Poland. The greater the number of traumatic experiences and victimization and the higher the severity of depressive symptoms, and frequency of non-suicidal self-injuries, the greater the severity of suicidal behaviour among adolescents in both the Polish and Ukrainian samples. These findings are consistent with results of the Ukraine Adolescent Mental Health Study [48, 49], as well as previous research [43] and theoretical models of adolescent suicidal behaviour [28].

Although risk factors, particularly traumatic experiences and mental health problems, explained most of the variability in adolescent suicidal behaviour, protective factors played a significant role, supporting the compensatory model of resilience as hypothesised. The results suggest that supportive parenting practices, a safe school environment, and a positive perception of the future have the strongest protective potential in the context of adolescent suicidal behaviour, followed by self-confidence. These findings highlight the importance of protective factors, including support and motivation systems that play a significant role in the positive development of adolescents. The results of our study are therefore consistent with Bronfenbrenner’s ecological model of human development [7] and factors included in multisystem perspective of resilience, such as family management, hope, optimism, belief in a better future, and positive perceptions of oneself and one’s family ([32], p. 533).

Furthermore, a positive perception of the future was found to be a significant protective factor associated with a lower risk of suicidal behaviour in adolescents. A Ukrainian study by Haletska et al., [19] found that high levels of resilience are closely linked to a future orientation and a positive perception of the present moment. It is precisely this focus on the future—seen as possible, valuable, and achievable—that helps overcome the effects of trauma and sustain mental resilience. In the context of war, the vision of the future becomes particularly sensitive—and at the same time crucial for maintaining mental health. Supporting and developing a positive perception of the future should be the goal of psychosocial interventions aimed at reducing the risk of suicide behaviours among young people.

Several significant interactions between protective factors and traumatic experiences support the protective resilience model, as hypothesized. Good child-mother communication (parental management) was found to moderate the relationship between exposure to traumatic experiences and suicidal behaviour. Teens who were exposed to traumatic experiences but maintained good communication with their parents (particularly their mothers) exhibited a lower risk of suicidal behaviour. Good communication with an adolescent child plays a key role in effective parenting, as it contributes to proper family conditions that allow parents to recognize the child’s stressful or traumatic experiences and help them to cope with such. In fact, family support and good communication with parents are probably the most well-known factors strengthening resilience in young people [31].

In addition, a sense of safety at school has also been found to moderate the relationship between traumatic experiences and suicidal behaviour. Adolescents exposed to traumatic experiences but supported by their school have been found to be at lower risk of engaging in suicidal behaviour. A sense of safety at school is one element of positive school climate, which has been confirmed in many studies to play a protective role in positive youth development [54].

The relationship between traumatic experiences and suicidal behaviour in adolescents was also moderated by self-confidence as measured by the PYD -VSF subscale. However, this effect was statistically significant only in the Polish and general samples and was not found to be statistically significant in the Ukrainian sub-sample. This most likely indicates that in conditions of active warfare, the protection of adolescents relies mainly on external resources, including, above all, the support of family and school. It is likely that, in wartime conditions, young people cannot rely solely on themselves and their personal competencies, but this interesting difference requires further clarification in research.

Our findings suggest that protective factors have less of an effect on suicidal behaviour than risk factors, and explain a smaller proportion of the variance in adolescent suicidal behaviour. The small scale of these effects may result from the inclusion of risk factors in the model, such as trauma, symptoms of depression and non-suicidal self-injuries, which are strongly associated with suicidal behaviour [28]. Given this situation, the small but significant effect of protective factors may have become impressive when such risk factors were first introduced into the hierarchical regression model. Notably, the effect size for the compensatory resilience model increases significantly when protective factors are introduced before risk factors into the regression model.

The results of this study indicate significant differences in suicidal behaviour among adolescents from Ukraine and Poland. Polish teens showed both a higher incidence and a higher risk of suicidal behaviour. The results also suggest that adolescents in Warsaw were more exposed to risk factors than their peers in Lviv and Drohobych. These results are consistent with other Polish-Ukrainian studies, which suggest that Ukrainian students and adults - despite higher levels of perceived danger - demonstrate better mental health than Polish students and adults [9, 22]). Despite the high level of distress caused by the war, the Ukrainian population demonstrates an increased sense of hope and belief in victory, as evidenced by the findings of this and other studies [19, 21]. We may therefore hypothesise that the lower level of suicidal thoughts and behaviours among Ukrainian adolescents, despite continuous exposure to traumatic conditions, may reflect the protective power of collective mobilisation, social cohesion, and national spirit, expressed through high levels of hope, trust, and belief in the future.

The finding of lower rates of suicidal behaviour among Ukrainian adolescents during wartime can be partly explained through E. Durkheim’s classical theory [11], which proposes that the level of suicide is inversely related to the degree of social integration. During major wars or political crises, when societies face a shared threat, the so-called “rallying around the flag” effect [35] may emerge. People unite in the response to a common danger, and under such conditions individuals tend to focus more on collective goals than on their own concerns, which helps prevent rumination about personal difficulties. This supports the assumption that the wartime context in Ukraine does not necessarily lead to higher vulnerability but may instead stimulate processes of collective mobilisation, social cohesion, and shared purpose.

Research by Lester [26] also confirms that suicide rates were relatively low during both World Wars. This helps explain why, despite the ongoing traumatic experience of war, Ukrainian adolescents show lower levels of suicidal thoughts and behaviours: the existential threat posed by the Russian invasion has strengthened national unity, which in turn serves as a powerful factor enhancing societal resilience.

From the perspective of the Interpersonal Theory of Suicide [12], the desire for death arises only when three factors co-occur: thwarted belongingness—a sense of profound loneliness and social disconnection; perceived burdensomeness—the belief that “others would be better off without me”; and an acquired capability for suicide—reduced fear of death resulting from repeated exposure to pain or trauma. In the context of war, the first two factors are significantly diminished: adolescents who volunteer, help their parents, or support friends do not feel like a burden—they feel needed. Nor do they feel isolated, as they perceive themselves to be part of a cohesive national community. The study confirmed that family resources and the school environment, as protective systems of social support that strengthen adolescents’ sense of belonging to a community, can buffer the negative effect of traumatic experiences on mental health.

However, the observed differences in the prevalence and risk of suicidal behaviour between the two study groups may also be due to differences in social and cultural factors. In Ukrainian society, the expression of psychological distress is still often stigmatised, especially among adolescents. Psychological difficulties are often perceived as a ‘personal weakness,’ which reduces the willingness of young people to talk openly about their emotional state, including suicidal thoughts. This may partly explain the lower frequency of reporting such thoughts among Ukrainian adolescents. In comparison, in Poland public policy encourages adolescents to search for professional help. This includes both awareness campaigns to reduce stigmatisation and specialised initiatives focused on the prevention of suicidal behaviour [40].

### Limitations and strengths

A limitation of this study is that the data are based on respondents’ self-assessment, which may potentially affect reliability. The study used a cross-sectional design, which precludes causal inferences. Thus, the relationships between the variables identified in our study are interpreted cautiously in terms of non-causal relationships. Furthermore, it should be noted that the tools used to study protective factors did not meet all of the highest methodological standards. The tools for examining three important protective variables (parental management, perception of the future, and sense of safety at school) were based on single questions, which carries the risk of less accurate measurement. Nevertheless, these three single questions yielded very promising results, so based on our experience, it is worth continuing the work on developing scales for more accurate measurement of the three protective factors. Another limitation of our study is that clustering by classroom or school was not examined. The model of standard hierarchical linear regression did not include such an option. Alternative analytical models suitable for analysing data with clustered structures, such as linear mixed models (LMM) or generalized linear mixed models (GLMM), do not provide such clear answers to our research hypotheses.

A more specific limitation of this study is that it was undertaken under the conditions of an ongoing war in Ukraine, which certainly had its impact on the overall condition of study participants and the research process itself. The difficulties in implementing the survey, such as airstrikes and power outages, highlight the challenging circumstances under which the data was collected. Despite these challenges, the research team successfully gathered a substantial amount of data, demonstrating their commitment to understanding adolescent mental health in both Poland and Ukraine.

*Generalizability.* Another significant limitation is the fact that in both cases the samples were not representative of their countries. Therefore, these results cannot be generalized to the adolescent populations in Poland and Ukraine.

## Conclusions

This study supports the usefulness of compensatory and protective models of resilience by showing that individual strengths, family resources, and a safe school environment can offset significant risk factors and moderate the negative effect of traumatic experiences on suicidal behaviour in adolescents. A specific implication of these findings is the need for targeted interventions that strengthen family and school support systems to reduce the risk of suicidal thoughts and behaviours among adolescents exposed to war-related threats and other risk factors.

## Supplementary Information

Below is the link to the electronic supplementary material.

Supplementary Material 1.

Supplementary Material 2.

Supplementary Material 3.

## Data Availability

The authors confirm that the data supporting the findings of this study are available within the article or its supplementary materials.
