# Supplementary material for: Suicidal behaviour and resilience-related factors among adolescents in Poland and war-affected Western Ukraine
Source: Child Adolesc Psychiatry Ment Health. 2026 Jun 28;20:136. doi: 10.1186/s13034-026-01126-0 (PMC13621684; doi:10.1186/s13034-026-01126-0)
Supplement: Supplementary file 1 [file 13034_2026_1126_MOESM1_ESM.docx]

Figure 1A. Relationship between exposure to traumatic experiences and the participant’s suicidal behaviour in the entire group of study participants for low, medium and high levels of family management (mother) : Low = 1 SD below, Medium = Mean, High = 1 SD above.

Figure 2A. Relationship between exposure to traumatic experiences and the participant’s suicidal behaviour in the entire group of study participants for low, medium and high levels of sense of safety at school: Low = 1 SD below, Medium = Mean, High = 1 SD above.

Figure 3A. Relationship between exposure to traumatic experiences and the participant’s suicidal behaviour in the entire group of study participants for low, medium and high levels of confidence (PYD -VSF subscale): Low = 1 SD below, Medium = Mean, High = 1 SD above.

Figure 4A. Relationship between exposure to traumatic experiences and the participant’s suicidal behaviour in the Ukrainian sample for low, medium and high levels of family management (mother): Low = 1 SD below, Medium = Mean, High = 1 SD above.

Figure 5A. Relationship between exposure to traumatic experiences and the participant’s suicidal behaviour in the Ukrainian sample for low, medium and high levels of sense of safety at school: Low = 1 SD below, Medium = Mean, High = 1 SD above.

Figure 6A. Relationship between exposure to traumatic experiences and the participant’s suicidal behaviour in Polish sample for low, medium and high levels of confidence (PYD -VSF subscale): Low = 1 SD below, Medium = Mean, High = 1 SD above.

Figure 7A. Relationship between exposure to traumatic experiences and the participant’s suicidal behaviour in the Polish sample for low, medium and high levels of sense of safety at school: Low = 1 SD below, Medium = Mean, High = 1 SD above.
