# Supplementary material for: Suicidal behaviour and resilience-related factors among adolescents in Poland and war-affected Western Ukraine
Source: Child Adolesc Psychiatry Ment Health. 2026 Jun 28;20:136. doi: 10.1186/s13034-026-01126-0 (PMC13621684; doi:10.1186/s13034-026-01126-0)
Supplement: Supplementary file 2 [file 13034_2026_1126_MOESM2_ESM.docx]

Table 1A. *Sociodemographic characteristics of the participants from Lviv/ Drohobych (Western Ukraine) and Warsaw (Poland)*

| **Variable** | **Categories** | **Ukrainian sample** | | **Polish sample** | | **Total** | |
| --- | --- | --- | --- | --- | --- | --- | --- |
|  |  | N | % | N | % | N | % |
| Gender | Female | 1158_a_ | 53.1 | 605_a_ | 52.4 | 1763 | 52.9 |
|  | Male | 946_a_ | 43.4 | 510_a_ | 44.2 | 1456 | 43.7 |
|  | Non-binary person | 75_a_ | 3.4 | 39_a_ | 3.4 | 114 | 3.4 |
| Age | 14 years old or younger | 1452_a_ | 66.6 | 552_b_ | 48.2 | 2004 | 60.3 |
|  | 15 years old or older | 727_a_ | 33.4 | 593_b_ | 51.8 | 1320 | 39.7 |
| Family  composition | With both parents | 1762_a_ | 80.9 | 833_b_ | 72.2 | 2595 | 77.9 |
|  | Only with mother | 251_a_ | 11.5 | 169_b_ | 14.7 | 420 | 12.6 |
|  | Only with father | 46_a_ | 2.1 | 21_a_ | 1.8 | 67 | 2.0 |
|  | With one parent and stepparent | 68_a_ | 3.1 | 82_b_ | 7.1 | 150 | 4.5 |
|  | With someone else | 52_a_ | 2.4 | 48_b_ | 4.2 | 100 | 3.0 |

*N* - number of respondents; *a, b* - letters indicate that the proportions of columns that are significantly different from each other at the .05 level

Table 2A. Prevalence of suicidal thoughts the last year, suicidal plans and attempts among adolescents in Lviv-Drohobych (Ukraine) and Warsaw (Poland) across gender

|  |  |  | *Have you ever had suicidal thoughts in the last 12 months?* | | | *Have you ever in your life seriously thought about taking your own life or planned how to do it?* | | |  | *Have you ever in your life tried to commit suicide?* | | |
| --- | --- | --- | --- | --- | --- | --- | --- | --- | --- | --- | --- | --- |
|  |  |  | Ukrainian sample  % | Polish sample  % | Total  % | Ukrainian sample  % | Polish sample  % | Total  % |  | Ukrainian sample  % | Polish sample  % | Total  % |
| Boys |  | Once or twice | 5.7 | 12.2* | 8.0 | 7.9 | 15.4* | 10.5 | Once | 2.3 | 4.9* | 3.2 |
|  |  | Sometimes | 2.7 | 5.5* | 3.7 | 1.8 | 4.3* | 2.7 | Two times | 0.4 | 2.9* | 1.3 |
|  |  | Often | 1.7 | 3.7* | 2.4 | 1.6 | 3.9* | 2.4 | Three times or more | 1.2 | 2.4 | 1.6 |
|  |  | At least once or twice | 10.1 | 21.4* | 14.1 | 11.3 | 23.6* | 15.6 | At least once | 3.9 | 10.2* | 6.1 |
| Girls |  | Once or twice | 10.3 | 21.3* | 14.0 | 14.9 | 22.6* | 17.5 | Once | 5.9 | 10.8* | 7.6 |
|  |  | Sometimes | 4.5 | 10.1* | 6.4 | 3.7 | 10.5* | 6.0 | Two times | 1.3 | 4.2* | 2.3 |
|  |  | Often | 2.2 | 10.8* | 5.2 | 1.7 | 11.1* | 4.9 | Three times or more | 2.0 | 5.5* | 3.2 |
|  |  | At least once or twice | 17.0 | 42.3* | 25.6 | 20.3 | 44.3* | 28.5 | At least once | 9.2 | 20.5* | 13.0 |
| Non-binary |  | Once or twice | 13.3 | 7.7 | 11.4 | 18.7 | 7.7 | 14.9 | Once | 9.3 | 7.7 | 8.8 |
|  |  | Sometimes | 9.3 | 5.1 | 7.9 | 6.7 | 15.4 | 9.6 | Two times | 2.7 | 23.1* | 9.6 |
|  |  | Often | 14.7 | 46.2* | 25.4 | 10.7 | 33.3* | 18.4 | Three times or more | 6.7 | 20.5* | 11.4 |
|  |  | At least once or twice | 37.3 | 59.0* | 44.7 | 36.0 | 56.4* | 43.0 | At least once | 18,7 | 51.3* | 29.8 |
| Total |  | Once or twice | 8.4 | 16.8* | 11.3 | 12.0 | 18.9* | 14.4 | Once | 4.5 | 8.1* | 5.7 |
|  |  | Sometimes | 3.9 | 7.9* | 5.3 | 3.0 | 7.9* | 4.7 | Two times | 1.0 | 4.3* | 2.1 |
|  |  | Often | 2.4 | 8.9* | 4.7 | 2.0 | 8.7* | 4.3 | Three times or more | 1.8 | 4.6* | 2.8 |
|  |  | At least once or twice | 14.7 | 33.6* | 21.2 | 16.9 | 35.5* | 23.4 | At least once | 7.2 | 17.0* | 10.6 |

*p<0.05

Table 3A. Prevalence of traumatic experiences among adolescents in Lviv-Drohobych (Ukraine) and Warsaw (Poland)

| ***Have you EVER had something really terrible /traumatic happen to you in your life?*** | Ukrainian sample  % | Polish sample  % | Total  % |
| --- | --- | --- | --- |
| Have you survived a flood, hurricane, earthquake? | 8.9 | 6.5* | 8.1 |
| Have you survived a major fire? | 3.9 | 4.2 | 4.0 |
| Have you survived a car accident or other serious accident? | 10.7 | 14.1* | 11.9 |
| Have you seen someone killed or seriously injured? | 16.0 | 34.1* | 22.2 |
| Have you ever been severely beaten or assaulted by someone? | 5.3 | 11.5* | 7.4 |
| Have you experienced sexual assault/sexual harassment | 5.3 | 9.4* | 6.7 |
| ***Have you been continuously exposed to traumatic experiences in the last year?*** |  |  |  |
| Serious conflicts at home (quarrels, fights, violence) | 12.9 | 27.6* | 17.9 |
| Violence, humiliation, ridicule or other harm from peers | 14.1 | 27.8* | 18.8 |
| Intimidation, blackmail by people around you or your neighbourhood | 4.8 | 16.0* | 8.6 |
| Sexual harassment | 3.5 | 6.5* | 4.5 |

* p<0.05

Table 4A. Comparison of risk and protective factors in the two samples (Ukrainian and Polish)

|  | M (SD) | |  |  |  | 95% CI for Cohen’s d | |
| --- | --- | --- | --- | --- | --- | --- | --- |
| Risk or protective factor | Ukrainian sample | Polish sample | t-test | p | Cohen’s d | Lower | Upper |
| 1. Traumatic experiences index | 0.80 (1.26) | 1.49 (1.60) | -12.59 | < 0.001 | -0.49 | -0.56 | -0.42 |
| 2. Victimization including violence at school and cyberbullying | 1.17 (0.43) | 1.38 (0.62) | -10.73 | < 0.001 | -0.43 | -0.51 | -0.36 |
| 3. Depressive symptoms | 1.86 (0.73) | 2.11 (0.83) | -8.71 | < 0.001 | -0.33 | -0.40 | -0.26 |
| 4. Non-suicidal Self-injury | 1.34 (0.84) | 1.94 (1.29) | -14.15 | < 0.001 | -0.58 | -0.66 | -0.51 |
| 5. Sensation seeking | 1.61 (0.84) | 2.01 (1.00) | -11.55 | < 0.001 | -0.44 | -0.52 | -0.37 |
| 6. Drug use | 1.04 (0.35) | 1.23 (0.77) | -7.95 | < 0.001 | -0.36 | -0.43 | -0.28 |
| 7. Tranquilizer use | 1.45(0.96) | 1.65 (1.23) | -4.79 | < 0.001 | -0.19 | -0.26 | -0.15 |
| 8. Family Management (Mother)) | 3.65 (1.21) | 3.26 (1.22) | 8.74 | < 0.001 | 0.32 | 0.25 | 0.39 |
| 9. Sense of safety at school | 3.15 (0.80) | 3.17 (.71) | 0.88 | 0.188 | 0.03 | -0.10 | 0.04 |
| 10. Confidence: PYD-VSF subscale | 3.55 (1.11) | 3.09 (1.07) | 11.76 | <0.001 | 0.42 | 0.35 | 0.49 |
| 11. Positive Perception of the Future | 7.43 (2.15) | 6.73 (2.49) | 8.12 | <0.001 | 0.31 | 0.24 | 0.38 |
